# Local Treatment of Ranula

**Published:** 1886-02

**Authors:** 


### Location and Treatment of Ranula.

Dr. E. Sonnenburg, in the *Archives fur Klinische Chirurgie*, xxix., 3, p. 627, treats the above subject. He relates the following case:

A six-year old girl showed, in a widely-opened mouth, a tumor, which fluctuated and was of a bluish color. It was situated in the floor of the mouth, and was elastic to the touch. This pressed the tongue upwards, and made speech and the taking of nourishment quite impossible. Wharton's duct was, on both sides of the tumor, free, visible, and readily sounded. The submental region was distended clear to the upper part of the thyroid cartilage. The extirpation was made through a six cm. long transverse incision in the region of the lower jaw. Still it was possible to free only the lower part of the cyst from the surrounding parts. On account of the great distension upwards, it was found necessary to evacuate the muco-purulent contents, and then through pulling forwards of the cyst walls, which causes the tongue also to follow, but finally freed the cyst from the soft parts. By means of examination by the finger it was found that the cyst was contained entirely in the substance of the tongue, and that only a thin shell of the muscles of the tongue was present. The bone substance of the lower maxilla was atrophic, and it projected past the superior maxillary three-fifths of an inch. On account of the falling back of the tongue, it was found necessary to draw this member forward with a thread. The wound, which was drained, healed in about fourteen days. The tongue appeared small, but in a normal condition, while the swallowing was good; but the speech was not yet distinct. The walls of the cyst showed a layer of epithelium. The contents consisted of mucus and pus cells.

The author has examined about fifty cases of ranula, and comes to the conclusion that they are in direct connection with the Blandin-Nuhn glands. These are two glands, situated at the apex of the tongue beneath the mucous membrane and the longitudinal muscular fibres formed by the styloglossus and longitudinalis inferior muscles.

In the operation, the author recommends the following proceeding: One inserts a curved needle into the cyst-wall above and parallel with the duct of Wharton, fixes the cyst-walls by means of threads, which, if possible, also fix the tongue substance; then make a cut parallel with the duct of Wharton, and dissect the anterior wall, which one can draw out by means of the thread. Cauterization or drainage are then unnecessary. Quite large cysts are best extirpated *in toto* from the regio-submentalis, which is not difficult.

In the author's fifty cases he observed no relapse.

